# Core temperature after birth in babies with neonatal encephalopathy in a sub‐Saharan African hospital setting

**DOI:** 10.1113/JP277820

**Published:** 2019-06-05

**Authors:** Christabel Enweronu‐Laryea, Kathryn A Martinello, Maggie Rose, Sally Manu, Cally J Tann, Judith Meek, Kojo Ahor‐Essel, Geraldine B Boylan, Nicola J Robertson

**Affiliations:** ^1^ Department of Child Health University of Ghana School of Medicine and Dentistry Accra Ghana; ^2^ Neonatal Intensive Care Unit Korle Bu Teaching Hospital Accra Ghana; ^3^ Institute for Women's Health University College London London UK; ^4^ Robinson Research Institute University of Adelaide Adelaide Australia; ^5^ Neonatology University College London Hospital NHS Foundation Trust London UK; ^6^ Maternal, Adolescent, Reproductive and Child Health Centre, Department of Infectious Disease Epidemiology London School of Hygiene and Tropical Medicine London UK; ^7^ INFANT Research Centre University College Cork Cork Ireland; ^8^ Department of Paediatrics & Child Health University College Cork Cork Ireland; ^9^ Division of Neonatology Sidra Medicine Doha Qatar

**Keywords:** Global health, hypoxic ischaemic encephalopathy, neonatal encephalopathy, therapeutic hypothermia, facilitated passive cooling

## Abstract

**Key points:**

- Therapeutic hypothermia (HT) to 33.0–34.0°C for 72 h provides optimal therapy for infants with neonatal encephalopathy (NE) in high‐resource settings. HT is not universally implemented in low‐ and middle‐income countries as a result of both limited resources and evidence.
- Facilitated passive cooling, comprising infants being allowed to passively lower their body temperature in the days after birth, is an emerging practice in some West African neonatal units.
- In this observational study, we demonstrate that infants undergoing facilitated passive cooling in a neonatal unit in Accra, Ghana, achieve temperatures within the HT target range ∼20% of the 72 h. Depth of HT fluctuates and can be excessive, as well as not maintained, especially after 24 h.
- Sustained and deeper passive cooling was evident for severe NE and for those that died.
- It is important to prevent excessive cooling, to understand that severe NE babies cool more and to be aware of facilitated passive cooling with respect to the design of clinical trials in low‐ and mid‐resource settings.

**Abstract:**

Neonatal encephalopathy (NE) is a significant worldwide problem with the greatest burden in sub‐Saharan Africa. Therapeutic hypothermia (HT), comprising the standard of care for infants with moderate‐to‐severe NE in settings with sophisticated intensive care, is not available to infants in many sub‐Saharan African countries, including Ghana. We prospectively assessed the temperature response in relation to outcome in the 80 h after birth in a cohort of babies with NE undergoing ‘facilitated passive cooling’ at Korle Bu Teaching Hospital, Accra, Ghana. We hypothesized that NE infants demonstrate passive cooling. Thirteen infants (69% male) ≥36 weeks with moderate‐to‐severe NE were enrolled. Ambient mean ± SD temperature was 28.3 ± 0.7°C. Infant core temperature was 34.2 ± 1.2°C over the first 24 h and 35.0 ± 1.0°C over 80 h. Nadir mean temperature occurred at 15 h. Temperatures were within target range for HT with respect to 18 ± 14% of measurements within the first 72 h. Axillary temperature was 0.5 ± 0.2°C below core. Three infants died before discharge. Core temperature over 80 h for surviving infants was 35.3 ± 0.9°C and 33.96 ± 0.7°C for those that died (*P* = 0.043). Temperature profile negatively correlated with Thompson NE score on day 4 (*r* ^2^ = 0.66): infants with a Thompson score of 0–6 had higher temperatures than those with a score of 7–15 (*P* = 0.021) and a score of 16+/deceased (*P* = 0.007). More severe NE was associated with lower core temperatures. Passive cooling is a physiological response after hypoxia–ischaemia; however, the potential neuroprotective effect of facilitated passive cooling is unknown. An awareness of facilitated passive cooling in babies with NE is important for the design of clinical trials of neuroprotection in low and mid resource settings.

## Key points

- Therapeutic hypothermia (HT) to 33.0–34.0°C for 72 h provides optimal therapy for infants with neonatal encephalopathy (NE) in high‐resource settings. HT is not universally implemented in low‐ and middle‐income countries as a result of both limited resources and evidence.
- Facilitated passive cooling, comprising infants being allowed to passively lower their body temperature in the days after birth, is an emerging practice in some West African neonatal units.
- In this observational study, we demonstrate that infants undergoing facilitated passive cooling in a neonatal unit in Accra, Ghana, achieve temperatures within the HT target range ∼20% of the 72 h. Depth of HT fluctuates and can be excessive, as well as not maintained, especially after 24 h.
- Sustained and deeper passive cooling was evident for severe NE and for those that died.
- It is important to prevent excessive cooling, to understand that severe NE babies cool more and to be aware of facilitated passive cooling with respect to the design of clinical trials in low‐ and mid‐resource settings.

## Introduction

Intrapartum‐related hypoxic events are a major cause of neonatal mortality and morbidity in low‐ and mid‐income countries (LMIC). Neonatal encephalopathy (NE) secondary to intrapartum events is estimated to affect 1.16 million babies per year, with the highest rates occurring in sub‐Saharan Africa (Lee *et al*. 2013). NE manifests with neurological dysfunction in the first days of life, including difficulty initiating and sustaining respiration, abnormal level of consciousness, depression of tone and reflexes, and, in many cases, seizures (Nelson & Leviton, 1991). Globally each year, NE is estimated to cause 287,000 deaths and over 50 million disability adjusted life years (Lee *et al*. 2013). NE is the second most common cause of avoidable childhood neurodevelopmental disability worldwide (Lawn *et al*. 2014). Reducing preventable neonatal death is a global Sustainable Development Goal (United Nations, 2015).

Ghana is a middle‐income country with ∼800,000 births per year. Intrapartum‐related hypoxic events account for around one‐third of all newborn deaths and are among the top 10 causes of all deaths in the country (WHO, 2016). NE is a major cause of disability in Ghanaian children (Adei‐Atiemo *et al*. 2015). During a 6 month period in 2015, birth asphyxia accounted for 30% of the 966 neonatal unit admissions at Korle Bu Teaching Hospital (KBTH) in Accra, Ghana (Samba, 2017). Twenty‐two percent of cases referred to KBTH with perinatal asphyxia died. Four out of five normal birth weight term deaths at KBTH neonatal unit were associated with intrapartum‐related hypoxic events.

Therapeutic hypothermia (HT) is standard clinical care for infants with moderate‐to‐severe NE in high‐income countries (National Institute for Health and Care Excellence, 2010). Cooling to 33.5°C for 72 h within 6 h of birth improves outcomes of death and neurodisability in the short (Edwards *et al*. 2010; Jacobs *et al*. 2013) and longer term (Guillet *et al*. 2012; Shankaran *et al*. 2012). In high income settings, the implementation of HT over the past decade has benefited patients, society and the economy (Azzopardi *et al*. 2012).

Despite the high burden of NE in Ghana and other LMIC, there is no readily available and affordable evidence‐based therapy for NE beyond supportive care. Modern technologies for providing HT and comprehensive monitoring of affected babies remain beyond the reach of many hospitals. A systematic review combining studies from LMIC settings suggested that HT was not associated with a reduction in neonatal mortality, although the confidence intervals were wide (Pauliah *et al*. 2013). Indeed, it is possible that HT may not be beneficial in low resource settings without sophisticated neonatal intensive care unit (NICU) care: a pilot study in a sub‐Saharan African hospital showed that cooling for 72h with water bottles was feasible but was associated with increased mortality, although the study was not powered for outcome measures (Robertson *et al*. 2008).

An emerging practice in the management of NE in low resource settings is to facilitate ‘passive cooling’. Passive cooling is known to occur in babies with NE in the first hours after birth; this was first described 60 years ago by Burnard and Cross (1958) and has more recently been observed during neonatal transport (Kendall *et al*. 2010), during HT in the NICU (Baumgart, 2008) and in the control arms of low‐income setting cooling studies (Robertson *et al*. 2008; Thayyil *et al*. 2013). Diverse methods are used in the management of intrapartum‐related NE in Ghanaian hospitals, including adapted low‐cost cooling technologies, ‘facilitated passive cooling’ or no intervention. It is not known whether facilitating passive cooling after perinatal hypoxia ischaemia is safe and beneficial or whether 72 h of controlled HT would be preferable in this setting. A better understanding of the endogenous temperature response in affected babies is needed to guide recommendations for the management of NE and the design of future HT trials in this setting.

The present study aimed: (i) to document the temperature profile of infants with moderate‐to‐severe NE over 80 h when undergoing typical standard management; (ii) to explore the relationship between temperature profile and NE severity and short‐term outcome; and (iii) to assess the difference in the ‘hypothermia dose’ with facilitated passive cooling compared to standard HT protocols typical of high resource settings.

We hypothesized that infants with NE undergoing facilitated passive cooling would demonstrate moderate transient HT.

## Methods

### Ethical approval

Ethical approval was obtained from the Institutional Review Board of Korle Bu University Teaching Hospital (KBTH) (KBTH‐IRB 00039/2017).

### Study design and participants

This prospective observational cohort pilot study was conducted in the neonatal unit of the Department of Child Health at KBTH, in Accra, Ghana. The study conformed to the standards set by the *Declaration of Helsinki*, except for registration in a database. Eligible infants were recruited between 19 June and 20 July 2017. Inclusion criteria were: gestation age ≥36 + 0 weeks, birth weight ≥2000 g, postnatal age <24 h and evidence of moderate‐to‐severe intrapartum‐associated NE. Moderate‐to‐severe intrapartum‐associated NE was defined as (i) evidence of intrapartum asphyxia (i.e. need for bag‐mask resuscitation at birth and Apgar score of <6 at 5 min of life) and (ii) either a Thompson NE assessment score of ≥7 or suspected clinical seizures. Infants in whom death was felt imminent and infants with major congenital malformations were excluded. Infants with swift clinical recovery following enrolment were subsequently excluded to allow discharge to the care of their mothers. In this pilot study, the recruitment of 10–20 infants was considered feasible during the study period (projected 30–60% recruitment rate, based on ∼30 NE admissions per month to KBTH neonatal unit in early 2017).

### Procedures

The local research team of selected doctors and nurses with experience in neonatal care were trained in the research procedures. Clinical care for participants was provided by the clinical team of KBTH neonatal unit and overseen by the attending consultants. Eligible infants were enrolled after stabilization by the clinical team and written informed consent (in either English or the local language) having been provided by the parents. Enrolled infants were transferred to one of four cot spaces in a side room reserved for study participants. The decision to use a side room for conducting the study was made by the nursing administration of the neonatal unit at KBTH aiming to minimize disruption to the rest of the neonatal unit. Temperature management and all aspects of clinical care were at the discretion of the clinical team and were not dictated by the study. Standard thermal care for term infants with NE at KBTH was facilitated passive cooling, with intermittent surface temperature monitoring. Infants were dressed in a nappy and nursed uncovered without an overhead heater in a room without fans or air‐conditioning. At a surface temperature below 34°C, standard practice was to provide warmth with light linen or blankets to prevent severe HT. The unit did not have the capacity to routinely monitor core temperature.

Trained medical research personnel completed daily brief neurological examinations for the first 4 days of life. These were used to determine a Thompson score (Thompson *et al*. 1997), comprising a validated tool for short‐term outcome prediction in NE infants in LMIC (Horn *et al*. 2013a; Horn *et al*. 2013b; Bhagwani *et al*. 2016), and a modified Sarnat encephalopathy stage (Shankaran *et al*. 2005).

Rectal (core), axillary and ambient air temperatures were continuously measured (every 1 min) and stored using a multichannel data logger (Squirrel SQ 2020; Grant Instruments, Cambridge, UK), from enrolment until 80 h of age. A nine French thermometer probe (FMT400; Metko Medikal, Ankara, Turkey) was inserted ∼3 cm into the rectum and taped in place. The rectal temperature probe was manually modified to interface with the data logger. This was inspected to ensure correct placement at regular intervals and on an *ad hoc* basis in case of aberrant readings, which were transcribed hourly. The axillary skin thermometer (Grant Instruments) was taped in the axilla. The ambient thermometer (Grant Instruments) was placed on the cot alongside the infant, exposed to the air.

Routine demographic, pregnancy and labour data were collected from the medical notes and by speaking with parents. Relevant data from the admission, including the method of temperature control and the length of hospital stay, were recorded. Infants were followed until discharge.

### Outcome measures

Short‐term outcome measures include survival to discharge and Thompson score on day 4 (0–6, normal–mild; 7–15, moderate; ≥16 or deceased, severe) (Horn *et al*. 2013a; Horn *et al*. 2013b).

### Statistical analysis

Data are summarized with counts (percentages) for categorical variables and as the mean ± SD for continuous variables. The rectal temperature data (collected every 1 min) was interrogated to exclude erroneous values and averaged over each hour. Mean rectal temperature over the first 24 h, as well as mean rectal, axillary and ambient temperature over the first 80 h, was calculated. The proportion of time (in minutes) over the first 72 h after birth that was within, below and above the target range for standard HT (33.0–34.0°C) was calculated as a percentage of measured time to account for variable age at commencement. Temperature values are reported as the mean ± SD. Using JMP, version 11.0.0 (SAS Institute Inc., Cary, NC, USA) and SPSS, version 22.0 (IBM Corp., Armonk, NY, USA), a *t* test or ANOVA with *post hoc* Fisher's least significant difference pairwise comparison was employed, as appropriate, to compare temperature data between short‐term outcome measures. A linear regression analysis was undertaken to evaluate the relationship between Thompson score on day 4 and temperature. In addition, using Prism, version 7.0 (GraphPad Software Inc., San Diego, CA, USA), Pearson's correlations were calculated for axillary compared with core temperature and to assess the impact of potential confounders, birth weight and phenobarbitone. Analysed data were normally distributed.

## Results

### Participants

**Figure 1:**
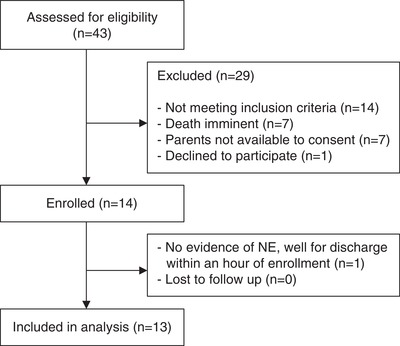
Eligibility flow chart Flow chart indicating the eligibility of the infants who were enrolled in the study.

During the 1‐month study period, 43 infants were assessed for eligibility and 14 were enrolled (Fig. 1). One infant was excluded within 1 h of enrolment as a result of rapid clinical improvement. Thirteen infants, with a mean gestation of 39 + 5 weeks (SD 12 days) and mean ± SD birth weight of 3138 ± 463 g were included in the analysis. The majority of infants had moderate NE (Thompson score 7–15) at enrolment (85%). Baseline characteristics are listed in Table 1.

**Table 1:** Baseline characteristics, short‐term outcome measures and clinical care during admission

Baseline characteristics and short‐term outcome (*n* = 13)	
Gestation (weeks)	39 ^+ 5 ^±^ ^1 ^+^ ^5^
Birth weight (g)	3138 ± 463
Male sex (*n*, %)	9 (69)
Apgar 1 min	3 ± 1.7
Apgar 5 min	4.9 ± 1.7
Thompson score at enrolment	11.3 ± 3
7–15 (n, %)	11 (85)
16+ (n, %)	2 (15)
Sarnat Grade at enrolment (*n*, %)	
Stage 2	10 (77)
Stage 3	3 (23)
Age at enrolment (h)	7.5 ± 7
Onset of labour (*n*, %)	
Spontaneous	10 (77)
Induction	2 (15)
LSCS not in labour	1 (8)
Mode of delivery (*n*, %)	
Vaginal	11 (85)
Emergency LSCS	2 (15)
Duration ROM (*n*, %)	
<18 h	7 (54)
≥18 h	4 (31)
Unknown	2 (15)
Resuscitation (*n*, %)	
Bag and mask IPPV	13 (100)
Intubation	0 (0)
Chest compressions	0 (0)
Day 4 Thompson score (*n*, %)	
0–6	6 (46)
7–15	4 (31)
16+	2 (15)
Deceased prior to day 4	1 (8)
Day 4 Sarnat grade (*n*, %)	
Normal/stage I	5 (38)
Stage II	4 (31)
Stage III	3 (23)
Deceased prior to day 4	1 (8)
Deaths (*n*, %)	3 (23%)
Day 4 Sarnat stage I	0/3
Day 4 Sarnat stage II	1/3
Day 4 Sarnat stage III	2/3
Overall length of hospital stay (days)	10 ± 4.6
Age at discharge home (days)	12 ± 3.8
Feeding at discharge (*n*, % of survivors)	
Exclusive breastfeeding	7 (70)
Breast and cup feeding	3 (30)
Maximal respiratory support (*n*, %)	
None	0 (0)
Oxygen by face mask or nasal prongs	9 (69)
Bubble CPAP	4 (30)
Suspected clinical seizures (*n*, %)	11 (85)
Received anti‐convulsants (*n*, %)^a^	10 (77)
Infection (*n*, %)	
Suspected clinical infection at admission	9 (69)
Received i.v. antibiotics	12 (92)
Received extended spectrum i.v. antibiotics	2 (15)
Blood culture positive (*n*/number sampled)	0/10

CPAP, continuous positive airway pressure (using heated humidified blended air and oxygen); LSCS, lower segment caesarean section; ROM, rupture of membranes; IPPV, intermittent positive pressure ventilation breaths.

a Anti‐convulsant therapy for all treated infants commenced with phenobarbitone (+/– subsequent phenytoin and midazolam). Values are the mean ± SD unless specified otherwise.

### Temperature profile

Standard thermal care with facilitated passive cooling was implemented for all infants. Temperature recordings commenced between 1.2 and 26 h of age (median 5 h, interquartile range 3–12 h). The mean ± SD ambient temperature in the nursery was 28.3 ± 0.7°C.

**Figure 2:**
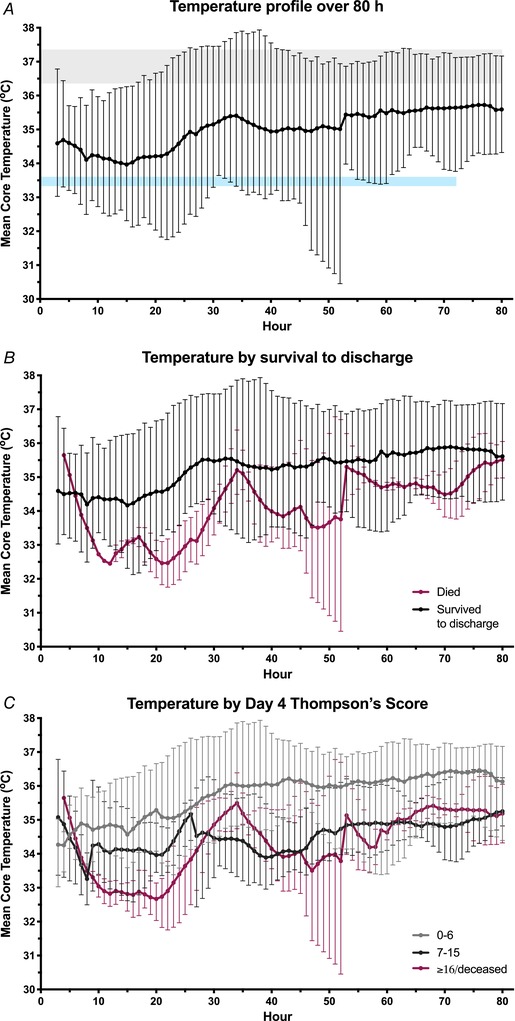
Core temperature profile over 80 h for infants undergoing facilitated passive cooling (mean ± range) *A*, rectal temperature of all infants over the first 80 h after birth. Passive HT is evident, with the nadir temperature at 15 h. The grey shaded area is the ‘normothermic’ range, whereas the blue line represents the target temperature for active therapeutic HT. Infants who died prior to discharge (*B*) and those with moderate and severe encephalopathy at day 4 (*C*) had deeper and more sustained HT. [Color figure can be viewed at wileyonlinelibrary.com]

The core temperature profile over 80 h for all infants is shown in Fig. 2 *A*. All infants were hypothermic during the study period. Mean core temperature over the first 24 h was 34.2 ± 1.2°C and, over 80 h, was 35 ± 1°C. The nadir mean core temperature was at 15 h of age. Over the first 72 h after birth, infants spent a mean ± SD 18 ± 14% (range 0–44%) of the measured time within the target temperature range for standard HT (33.0–34.0°C); core temperature was <33.0°C for 11 ± 18% of the time and >34°C for 71 ± 22% of the time (Table 2). Seven infants (54%) had temperature recordings less than 33°C, of which four were between 32.1 to 32.5°C and one was below 32°C. The one infant who did not reach 33.5°C (nadir temp 35.1°C at 10.5 h) went on to develop fever and an elevated C‐reactive protein of 350 mg L^–1^ and was treated with extended‐spectrum i.v. antibiotics for suspected sepsis.

**Table 2:** Proportion of time within the first 72 h after birth that was within, below and above the target range for standard HT (33.0–34.0°C)

	Overall	Mortality			Day 4 Thompson score			
		Survived	Died		0‐6	7–15	16+/ deceased	
	Mean ± SD	Mean ± SD	Mean ± SD	*P* value	Mean ± SD	Mean ± SD	Mean ± SD	*P* value
Time measured during 1 s at 72 h after birth (h)	63.4 ± 7.6	65.0 ± 6.0	58.0 ± 9.4	0.19	64.1 ± 7.4	61.8 ± 9.5	64.0 ± 3.8	0.905
Proportion within HT target range (33.0–34.0°C) (%)	17.7 ± 13.8	15.1 ± 13.0	26.4 ± 12.9	0.25	6.7 ± 5.9	29.7 ± 12.3	23.6 ± 9.1	**0.015**
Proportion time below 33.0°C (%)	11.0 ± 17.9	5.8 ± 6.8	28.4 ± 29.1	0.39	5.3 ± 7.8	5.2 ± 3.4	30.1 ± 27.9	0.125
Proportion time above 34°C (%)	71.3 ± 21.8	79.1 ± 14.4	45.2 ± 21.9	**0.015**	88.0 ± 8.4	65.0 ± 12.0	46.3 ± 22.1	**0.010**

Values are presented as overall data and are separated by short‐term outcome measures (mortality and day 4 Thompson score). Time is represented as a percentage of time to account for variable commencement time. *P* values are from an independent *t* test (mortality) and an ANOVA (Thompson score). *P* values < 0.05 are shown in bold.

The depth of HT achieved by these infants had not been previously recognized because the neonatal unit lacked the capacity to measure core temperature. To ensure patient safety, the attending consultant amended the temperature management care plan to include the lower core temperatures recorded by the study thermometers. This amendment was core temperature <33.5°C: apply one blanket; core temperature <33.2°C: apply a second blanket; and core temperature <33.0°C: place infant in an incubator. Five infants had blankets applied and one was warmed in an incubator. Four infants received heated humidified continuous positive airway pressure for respiratory support.

Axillary skin temperature was 0.5 ± 0.2 °C below core rectal temperature (range 0.1–1 °C). There was a close correlation between mean core and axillary temperature over 80 h (Pearson's *r* ^2^ 0.96, *P* < 0.0001).

### Short‐term outcome

Short‐term outcomes are listed in Table 1. By day 4, Thompson score had improved to 0–6 for six (46%) infants; four (30%) infants had persisting moderate encephalopathy (score 7–15), two were severe (score 16+) and one infant was deceased. Three infants (23%) died before discharge: one with moderate NE and two with severe NE. All surviving infants were discharged home.

### Relationship between temperature profile and outcome

The differences in temperature profile over 80h by outcome are illustrated in Fig, 2 *B* and *C*. The 80 h mean core temperature of infants who died prior to discharge was 33.96 ± 0.7°C, cooler than 35.3 ± 0.9°C for those that survived [mean difference 1.4°C, 95% confidence interval (CI) = 0.1–2.7, *P* = 0.043]. The minimum core temperature was also lower for those that died (31.7 ± 0.9°C) compared to those that survived (33.3 ± 0.8°C) (mean difference 1.6°C, 95% CI = 0.3–2.9, *P* = 0.022).

The mean core temperature over 80 h for infants with a day 4 Thompson score of 0–6 was 35.8 ± 0.7°C. This was higher than those infants with a day 4 score of 7–15 (34.5 ± 0.5°C) or 16+/deceased (34.0 ± 0.7°C) (mean difference 1.3°C, 95% CI = 0.2–2.4, *P* = 0.021 and mean difference 1.8°C, 95% CI = 0.6–2.9, *P* = 0.007, respectively). The minimum core temperature for the 16+/deceased infants was also lower than that for those infants with a Thompson score of 0–6 (mean difference 2.0°C, 95% CI = 0.8–3.3°C, *P* = 0.004). This was despite the 16+/deceased group being exposed to a higher mean ambient temperature (mean difference 1.2°C, 95% CI = 0.3–2.2°C, *P* = 0.014).

**Figure 3:**
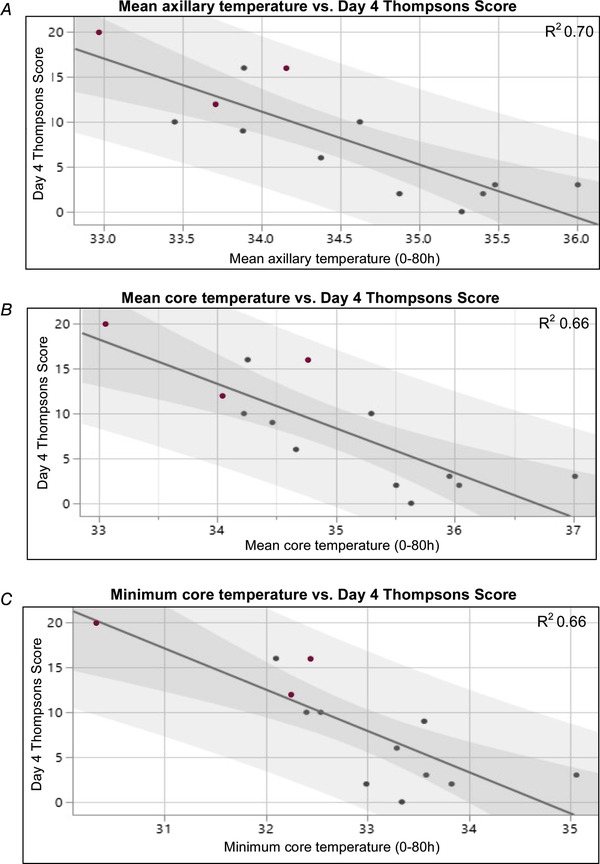
Relationship between day 4 Thompson score and infant temperature Linear regression model showing relationship between day 4 Thompson score and mean axillary (*A*), mean core (*B*) and minimum core (*C*) temperature. There were strong negative correlations between each of these measures (*r* ^2 ^≥ 0.66). The dark shading represents the 95% CI of the mean and the lighter shading indicates the 95% prediction interval. Infants who died prior to discharge are indicated with a burgundy dot. [Color figure can be viewed at wileyonlinelibrary.com]

Day 4 Thompson score demonstrated a strong negative correlation with mean axillary (*r* ^2^ = 0.71, *P* = 0.0004), mean core (*r* ^2^ = 0.66, *P* = 0.0007) and minimum core (*r* ^2^ = 0.66, *P* = 0.0008) temperature (Fig. 3). For these calculations, the one infant who had died prior to day 4 was assigned a Thompson score of 20 out of a possible 22, which is consistent with their score prior to death.

Infants with a day 4 Thompson score of 7–15 or 16+/deceased spent a greater proportion of the first 72 h within the ‘therapeutic hypothermia’ target range compared to infants who scored 0–6 (*P* = 0.006 and 0.043, respectively) (Table 2). Infants who scored 0–6 on day 4 spent a greater proportion of time above 34°C (*P* = 0.048 and 0.004 compared to 7–15 and 16+/deceased, respectively), as did infants that survived to discharge compared to those that died (*P* = 0.015). There was a strong positive correlation between day 4 Thompson score and the proportion of time spent ≤34 °C in the first 72 h (Pearson's *r* ^2^ = 0.72, *P* < 0.001).

There was no correlation between day 4 Thompson score and birth weight (Pearson's *r* ^2^ = 0.1). There was no correlation between core temperature and either birth weight (Pearson's *r* ^2^ = 0.3) or total phenobarbitone dose kg^–1^ (Pearson's *r* ^2^ = 0.3).

## Discussion

In this pragmatic, prospective, observational cohort study of term babies with moderate to severe NE in a hospital setting in Ghana, infants were hypothermic during the first 80 h after birth, even without active induction of HT (standard care in NICUs typical of high‐resource settings) (National Institute for Health and Care Excellence, 2010). Temperature management did not include a cooling device; infants were undressed and covered with a light blanket in an ambient temperature of 28°C. Core temperature was within the target range for ‘therapeutic hypothermia’ (33–34°C) for an average of 18% of the first 72 h and <33°C for a further 11% of measurements captured. The nadir temperature occurred at ∼15h after birth; most infants recorded core temperatures ≤33.5°C. The depth of facilitated passive cooling in the first 80 h correlated with short‐term outcome of death and the severity of NE.

Facilitated passive cooling is a pragmatic way to try to optimally manage these babies, as well as cope with the large number of admissions with NE, staff shortages and equipment limitations. Passive cooling in newborns following perinatal hypoxic ischaemic events was first described by Burnard and Cross (1958) who noted that NE infants had lower core temperatures than non‐asphyxiated babies for the first 16 h after birth. Similarly, Dawkins and Hull (1964) reported a loss of thermogenesis in rabbit pups during and subsequent to a period of hypoxia. More recently, passive cooling was documented in infants randomized to the control arm of feasibility cooling studies in hospital settings in Uganda and India (Robertson *et al*. 2008; Thayyil *et al*. 2013). Indeed, during standard HT using a servo‐controlled water mattress in high resource NICU settings, the water temperature has typically been observed to be higher than the infant core temperature (i.e. the infant often needs ‘warming’ to 33.5°C, particularly in the first 12 h) (Baumgart, 2008). This period of passive cooling reflects a loss or alteration of thermoregulation. Usual neonatal responses to cold are both behavioural (e.g. limb flexion to reduce exposed surface area and crying to attract maternal attention) and autonomic [e.g. vasoconstriction and non‐shivering thermogenesis within brown adipose tissue (BAT)] (Jayasinghe, 2015). Newborn infants at term age have a high proportion of BAT, which enables them to endure cold stress after birth (Aherne & Hull, 1966). Recognition of cold stimuli by peripheral and central control centres may be impaired and behavioural responses to cold may be lessened by NE. A lack of BAT thermogenesis following asphyxia may be the result of a loss of energy substrate and altered central control of thermoregulation. Given the known neuroprotective benefits of 72 h of controlled HT, it may be an adaptive survival mechanism. Indeed, passive cooling in response to hypoxia is evolutionary conserved throughout many species, suggesting a survival advantage (Steiner & Branco, 2002).

The severity of NE correlated strongly with the degree of passive cooling, demonstrating a greater alteration in thermoregulation in these infants. A loss of BAT activity is probably central to this. In a cohort of NE infants undergoing HT in a high resource setting, those infants with the least need for active cooling intervention to maintain target temperature had the lowest BAT activity (Carlisle *et al*. 2015b); those same infants had a greater probability of showing evidence of brain injury on MRI compared to infants who required more active cooling (Carlisle *et al*. 2015a). In combination with our findings, this supports the idea that those infants who are ‘easy to cool’ are probably more severely affected, at the same time suggesting that a higher core temperature might be a good prognostic indicator.

The depth of cooling observed in the present study of facilitated passive cooling was unexpected, particularly for those infants with severe NE. Almost all of the infants demonstrated a core temperature of 33.5°C or less during the first 80 h after birth. Importantly, a number of infants had core temperatures <32.5°C. Indeed, cooling to this depth may be hazardous, with an increased risk of mortality and persistent pulmonary hypertension and no improvement in outcome at 2 years being seen in a clinical trial of deeper and longer cooling (Shankaran *et al*. 2014; Shankaran *et al*. 2017). Standard HT (33.5°C for 72 h) in sophisticated NICU settings is associated with bradycardia and thromobocytopenia (Jacobs *et al*. 2013). NICU facilities to manage complications of overcooling are not currently available at KBTH, as is probably also the case for many neonatal units throughout the world. Given the available evidence regarding the risks of deep HT, the standard thermal care plan was amended during the present study. This resulted in the enrolled infants being warmed by blankets or incubators to 33.5°C. The use of an incubator accounts for the increased ambient temperature observed for infants with severe NE. Affordable and safe technologies for continuous monitoring of core temperature in infants undergoing facilitated passive cooling may reduce the risk of overcooling and related complications in mid‐ and low‐resource settings.

Pre‐clinical studies have been important with respect to understanding the dose–response of HT. There appears to be the requirement of a critical brain and body temperature below 34°C for effective brain protection. For example, in the fetal sheep undergoing head cooling from 90 min after ischaemia, neuroprotection was seen only in fetuses that had a sustained fall of the extradural temperature to <34°C (Gunn *et al*. 1997). In the piglet model, there is a clear U‐shaped response of brain cell death and core temperature after hypoxia ischaemia; a core temperature reduction of >5°C was associated with increased cell death and inflammation compared to cooling by 3.5–5°C (Alonso‐Alconada *et al*. 2015). It is difficult, however, to extrapolate precise temperatures from these pre‐clinical models because their core temperatures are higher than humans. Regarding duration of cooling, in the sheep model, 72 h of cooling provided optimal protection (Davidson *et al*. 2018a; Davidson *et al*. 2019) and the rate of rewarming was of less importance (Davidson *et al*. 2018b). Stopping cooling at 48 h but not 72 h in the fetal sheep was associated with progressive deterioration of the amplitude integrated electroencephalogram. However, HT maintained for ≤24h has proved neuroprotective in rodent and porcine models (Thoresen *et al*. 1995; Alonso‐Alconada *et al*. 2015). Preventing passive cooling responses to hypoxia in a rodent model resulted in an increase in mortality and brain injury (Reinboth *et al*. 2016). The implications of these findings for facilitated passive cooling in settings without access to sophisticated NICU care are unclear, such that it is not known whether a short period of variable cooling provides brain protection, although temperatures below 32.5°C are probably not beneficial.

There are some limitations to the present study. This was intended to be an observational study of usual practice; however, core temperature data were made available to the clinical team to ensure safety of enrolled infants. The availability of continuous core data, rather than intermittent surface temperature data, and the amendment to the temperature management plan influenced standard thermal care. This will have resulted in higher mean temperature recordings for some infants and therefore the depth of HT achieved with facilitated passive cooling was probably underestimated. In addition, enrolled infants were cared for in a separate room by dedicated research staff, aiming to avoid disruption to the rest of the neonatal unit, which may have impacted their care. Long‐term outcome measures are not included in the present study.

There may have been other contributors to the observed core temperature reductions in addition to facilitated passive cooling post NE. In severely encephalopathic infants, ongoing postnatal hypoxia as a result of apnoea and airway compromise may have contributed. Phenobarbitone, which was administered to 77% of infants, can cause HT at toxic levels. In an NE cohort, the use of phenobarbitone was associated with a modestly lower core temperature (Sant'Anna *et al*. 2012). However, this observation is possibly a result of the severity of brain injury and the resulting seizure frequency, rather than a phenobarbitone effect. Phenobarbitone dose was not associated with core temperature in our cohort. Intrauterine growth restriction is associated with an increased risk of NE (Badawi *et al*. 1998), as well as postnatal HT and reduced BAT stores (Aherne & Hull, 1966). In our cohort, birth weight was not correlated with mean core temperature or Thompson score and also does not account for the strong relationship between Thompson score and temperature. Infection and inflammation are known contributors to brain injury and encephalopathy and it is possible that HT may be less efficacious in the setting of infection (Osredkar *et al*. 2014; Martinello *et al*. 2018). In our cohort, no infants had culture positive sepsis; however, blood cultures were taken after commencing antibiotics for some infants. Twelve infants received antibiotics, whereas two of these went on to extended spectrum antibiotic coverage as a result of a strong clinical suspicion of sepsis. One of these infants did not demonstrate passive cooling beyond 35°C and later was febrile. It is possible that infection/inflammation without a hypoxic event was the cause of this infant's encephalopathy or, alternatively, that co‐existing infection interfered with facilitated passive cooling.

The benefit of facilitated passive cooling is unknown. Althogh the infants in the present study reached temperatures in the target range of HT, these fluctuated and, on average, were not maintained for more than 20% of the first 72 h. Passive cooling was most evident in the first 24 h, with many infants being above the target HT range on days 2 and 3. The available preclinical and clinical evidence suggests that cooling to 33.5°C for 72 h is most beneficial in the NICU setting (Gunn *et al*. 2017). However, it is plausible that these shorter periods of moderate passive cooling might confer some neuroprotective benefit. We suggest that active warming of infants with moderate to severe NE in low‐ and mid‐resource settings to achieve ‘normothermia’ may be detrimental and unethical until more information is obtained. An awareness of facilitated passive cooling in babies with NE in low‐ and mid‐resource settings is important for the design of clinical trials of neuroprotection in such settings where the burden of NE is high. However, facilitated passive cooling may not be sufficiently stable to be used as a control arm in clinical trials assessing adjunct therapies or HT itself.

For neonatal units in low‐ and mid‐resource settings considering the implementation of facilitated passive cooling, we advise the need for temperature monitoring, a basic level of neonatal care and dedicated nursing staff for infants in the acute stages of NE to avoid secondary brain injury. Careful monitoring of the core temperature is essential to ensure that excessive cooling does not occur, particularly for infants with severe NE.

## Additional information
